# Natural language processing to extract symptoms of severe mental illness from clinical text: the Clinical Record Interactive Search Comprehensive Data Extraction (CRIS-CODE) project

**DOI:** 10.1136/bmjopen-2016-012012

**Published:** 2017-01-17

**Authors:** Richard G Jackson, Rashmi Patel, Nishamali Jayatilleke, Anna Kolliakou, Michael Ball, Genevieve Gorrell, Angus Roberts, Richard J Dobson, Robert Stewart

**Affiliations:** 1 Institute of Psychiatry, Psychology & Neuroscience, King's College London, London, UK; 2 Department of Computer Science, University of Sheffield, Sheffield, UK

**Keywords:** Natural Language Processing, Serious Mental Illness, Symptomatology, MENTAL HEALTH, clinical informatics

## Abstract

**Objectives:**

We sought to use natural language processing to develop a suite of language models to capture key symptoms of severe mental illness (SMI) from clinical text, to facilitate the secondary use of mental healthcare data in research.

**Design:**

Development and validation of information extraction applications for ascertaining symptoms of SMI in routine mental health records using the Clinical Record Interactive Search (CRIS) data resource; description of their distribution in a corpus of discharge summaries.

**Setting:**

Electronic records from a large mental healthcare provider serving a geographic catchment of 1.2 million residents in four boroughs of south London, UK.

**Participants:**

The distribution of derived symptoms was described in 23 128 discharge summaries from 7962 patients who had received an SMI diagnosis, and 13 496 discharge summaries from 7575 patients who had received a non-SMI diagnosis.

**Outcome measures:**

Fifty SMI symptoms were identified by a team of psychiatrists for extraction based on salience and linguistic consistency in records, broadly categorised under positive, negative, disorganisation, manic and catatonic subgroups. Text models for each symptom were generated using the TextHunter tool and the CRIS database.

**Results:**

We extracted data for 46 symptoms with a median F1 score of 0.88. Four symptom models performed poorly and were excluded. From the corpus of discharge summaries, it was possible to extract symptomatology in 87% of patients with SMI and 60% of patients with non-SMI diagnosis.

**Conclusions:**

This work demonstrates the possibility of automatically extracting a broad range of SMI symptoms from English text discharge summaries for patients with an SMI diagnosis. Descriptive data also indicated that most symptoms cut across diagnoses, rather than being restricted to particular groups.

**Strengths and limitations of this study**

- The number and diversity of symptomatology concepts that we successfully modelled indicates that this task is suitable for natural language processing.
- The large number of records in the Clinical Record Interactive Search database gives insight into the reporting realities of symptomatology in a typical UK National Health Service Mental Health Trust for individuals who have received an International Classification of Diseases, Tenth Revision, severe mental illness (SMI) diagnosis.
- Our negative control group suggests a wide under-reporting of SMI symptoms in patients who have not received an SMI diagnosis, although our models were not validated in this group and such patients may have later received an SMI diagnosis after our analysis was concluded.
- Similarly, our models were validated on English text from a single UK site—the models may not generalise across different institutions and geographic/medical dialects.
- We did not attempt to resolve temporal aspects of symptomatology in this study, which will be necessary for future predictive modelling approaches.

## Introduction

### EHRs in health research

Electronic health records (EHRs) are recognised as a valuable source of data to support a wide range of secondary informatics use cases, such as decision support, observational research and business intelligence.^1^ With appropriate handling, EHRs may be able to overcome the cost barriers to generating sufficient data for addressing complex questions that would be out of reach for more conventional patient recruitment protocols.^2–4^ However, the use of EHRs in this way is known to create a range of new issues that need to be addressed before the data can be considered of sufficient quality suitable for research.^5^

### Symptomatology of severe mental illness

In mental health research and clinical practice, it is often argued that the symptoms expressed by a patient in the course of their illness represent a more useful description of the disorder and indications for intervention than the concept of a diagnosis.^6^ ^7^ While common conditions in mental health are represented in classification taxonomies such as the International Classification of Diseases (ICD) and Diagnostic and Statistical Manual(DSM) systems, generally speaking, it is the symptomatology of a condition that is used by clinicians to determine an appropriate treatment plan. This is due to the broad symptomatic manifestations of mental disorders, in the sense that, at a given time, a patient assigned a diagnosis (such as schizophrenia) can present with all, many or very few of the symptoms associated with the condition. This is particularly pertinent to clinical practice where diagnoses are not necessarily assigned using research criteria. The problems of diagnostic semantics are especially apparent in severe mental illness (SMI; schizophrenia, schizoaffective disorder and bipolar disorder). Here, the controversy is compounded by the high frequency of mental health comorbidities and shortcomings in our current understanding of the biological underpinnings of mental disorders, which in turn limit our ability to subclassify the conditions. For example, Van Os *et al*^8^ suggest that there are overlapping genetic, neurobiological and clinical features between different categories of mental disorder, and Insel *et al*^9^ suggest that within each diagnostic category there is a considerable degree of heterogeneity and that the diagnostic category in itself provides little information about future clinical outcomes. In addition, the lack of genetic and other objective tests for many mental disorders has led to a requirement for detailed, interpersonal observation of patients, cumulating in pragmatic symptomatology-based assessments.^10–14^ Information on specific symptoms is typically recorded in unstructured parts of the EHR,^15^ and the incorporation of structured instruments for recording symptoms has not so far proved feasible in routine clinical practice outside specialist services. Hence, the free text portion of the mental health EHR contains a potentially vast and complex tapestry of clinical information which to date has been effectively ‘invisible’ when it comes to the generation of data for administration, business intelligence, research or clinical evaluation.

Such a situation thus represents a quandary for mental health informaticians and clinical researchers alike. A common task in health research is to group patients with similar conditions into appropriate cohorts, which will almost inevitably require ascertaining common factors pertinent to their disorder.^16–18^ Diagnoses form semantically convenient units, although the usefulness may be disputed and/or lacking in granularity. Symptomatology may offer more objective, relevant groupings but the data may be locked in unstructured free text, presenting unique data extraction problems.

### Natural language processing and information extraction

Natural language processing (NLP) and its subdiscipline of Information Extraction (IE) are commonly employed within clinical records to process large quantities of unstructured (human authored) text and return structured information about its meaning.^19–21^ Medical entities frequently targeted include medications, diagnoses, smoking status and other factors influencing risk, course or outcome for disorders of interest.^21^ ^22^ A large number of tools and frameworks exist for general purpose information extraction from clinical dictionaries, such as cTAKES,^22^ NOBLE^23^ and MedLee.^24^ However, there has been little application of NLP techniques in mental healthcare data despite the volumes of text-based information contained here, and even less on ascertaining symptomatology. Here, we introduce the CRIS-CODE project, which has the long-term objective of offering comprehensive NLP models for mental health constructs. The focus of the initial programme of work described here was to develop sentence classification models for a substantial range of SMI symptomatology, to allow automatic extraction for many of the most informative symptoms from the patient narrative. It is envisaged that the outcomes will support a range of future research and clinical applications.

## Materials and methods

### Corpus selection and preprocessing: the South London and Maudsley Mental Health Case Register

The South London and Maudsley NHS Foundation Trust (SLaM) is one of the largest mental healthcare organisations in Europe, and provides mental health services to 1.2 million residents in its geographic catchment of four south London boroughs (Lambeth, Southwark, Lewisham and Croydon), in addition to national specialist services. SLaM adopted fully EHRs for all its services during 2006, importing legacy data from older systems during the process of assembly. In 2007–08, the Clinical Record Interactive Search (CRIS) application was developed with funding from the British National Institute for Health Research.^25^ CRIS generates a research database consisting of a pseudonymised version of SLaM's EHR system: currently containing de-identified patient records on more than 250 000 patients and over 3.5 million documents in common word processor formats. Since its development, the data contained have been substantially enhanced through external linkages and NLP.^26^ Patient consent was not required for this retrospective study.

### Definitions of SMI symptoms

A keyword lexicon of SMI symptoms was defined by a team of psychiatrists, based on pragmatic criteria. First, the potential salience of symptoms for research applications was considered, particularly their incorporation in symptom scales in common clinical use, such as the Positive and Negative Symptoms Scale (PANSS)^13^ and Young Mania Rating Scale (YMRS)^27^ which were used as templates for guidance. Second, the language used in routine clinical records was taken into consideration in choosing symptoms, focusing particularly on those which were likely to be recorded in the most consistent and tractable language, based on clinical experience. Third, we sought a priori to extract sufficient numbers of symptom types to generate scales for further evaluation within the following five domains: (1) positive symptoms; (2) negative symptoms; (3) disorganisation symptoms; (4) manic symptoms and (5) catatonic symptoms. The first four of these followed the findings of Demjaha *et al*,^28^ although we had not at this point attempted to define depressive symptoms. Catatonic symptoms were further added to improve consistency with the study of Cuesta and Peralta,^29^ and as a symptom group of interest, which is often not adequately captured in dimensional studies because of its relative rarity in recruited clinical samples.

We defined the NLP task as a sentence classification problem, with a classifiable instance as a sentence containing a symptom keyword or the general constructs of ‘negative symptoms’ or ‘catatonic syndrome’ (referring to groups 2 and 5 above). In addition to the keywords, clinically relevant modifier terms were also defined for some concepts, in order to produce subclassifications of symptoms where appropriate (table 1). If a modifier term was detected within eight words of a keyword, the modifier was deemed to be a possible relation. We further specified that modifiers could be ‘mandatory’ (meaning a modifier was required to be present for our definition of an instance to be met), or ‘optional’ (meaning only the keyword needed to be present for our instance definition to be met) (table 2). Regarding potential biases that might result from missing synonyms outside of our selected keywords, we did not consider this to be a significant problem. Clinical staff receive substantial training about how to document symptomatology in specific ways, in order to differentiate between a clinical opinion (‘the patient exhibited apathy’) and a non-clinical opinion (‘the patient expressed indifference towards their treatment today’), and therefore chose our keywords in line with the standard methods of symptom documentation to avoid uncertainty in the authors intent. Similarly, our objective was to identify clinician-assigned constructs, rather than attempt to classify descriptions of experiences—for example, identifying the recorded assignment of ‘hallucination’ as a symptom, rather than the description of the person's perceptual disturbance; identifying the recording of ‘delusion’ rather than the description of the false belief.

**Table 1:** Symptom instance definitions

SMI concept	Keyword strings	Modifier strings	Lax or strict modifiers	SNOMED-CT (SCTID)†
Aggression	aggress*			61372001
Agitation	agitat*			106126000
Anhedonia	anhedon*			28669007
Apathy	apath*			20602000
Arousal	arous*			(none)
Blunted or flat affect	Affect	blunt*, flat*, restrict*	Optional	6140007/932006/39370001
Catalepsy	catalep*			247917007
Catatonic syndrome	catatoni*			247917007
Circumstantial speech	circumstan*			18343006
Deficient abstract thinking	Concrete			71573006
Delusions	delusion*			2073000
Derailment of speech	derail*			65135009
Diminished eye contact	eye contact			412786000
Disturbed sleep	Sleep	not, poor*, interrupt*, nightmare*, disturb*, inadequat*, disorder*, prevent*, stop*, problem*, difficult*, reduced*, less*, impair*, erratic*, unable*, worse*, depriv*	Optional	26677001
Echolalia	Echolalia			64712007
Echopraxia	Echopraxia			33184005
Elation	elat*			34822003
Elevated mood	Mood	elevat*	Mandatory	81548002
Emotional withdrawal	withdraw*			247755007
Euphoria	euphor*			85949006
Flight of ideas	flight of idea			28810003
Formal thought disorder	Ftd thought disorder			41591006
Grandiosity	grandios*			247783009
Hallucinations	hallucinate*	audit*, visual*, olfact*, tactil*, third person, first person, 3rd person, 1st person,	Optional	45150006/64269007/39672001/66609003/277533007/
Hostility	hostil*			79351003
Immobility	immobil*			404975000
Insomnia	insom*			193462001
Irritability	irritabl*			55929007
Loosening of associations	associat*			55346003
Loss of coherence	coheren*			284596004
Low mood	Mood			366979004
Mannerisms	Mannerism*			248026005
Mutism	Mute Mutism			88052002
Negative syndrome	negative symptom*			(none)
Paranoia	paranoi*			191667009
Persecutory ideation	persecu*			216004
Perseverance	persever*			44515000
Poor motivation	motivat*			26413003
Poor rapport	Rapport			710497003
Posturing	postur*			271694000
Poverty of speech	speech*	poverty*, impoverish*	Mandatory	72123004
Poverty of thought	poverty of thought			56435009
Pressured speech	speech*	pressure*	Mandatory	53890003
Rigidity	rigid*			311535006
Social withdrawal	withdraw*	social*	Mandatory	105411000
Stereotypy	stereotyp*			84328007
Stupor	stupor*			89458003
Tangential speech	tangent*			74396008
Thought block	though* block			2899008
Waxy flexibility	Waxy			13052006

†Best matches in SNOMED-CT, UK-edition v20160401.

SMI, severe mental illness; SNOMED, Systematized Nomenclature of Medicine; SNOMED-CT (SCTID), Systematized Nomenclature of Medicine—Clinical Terms Identifier.

**Table 2:** Examples of instances

Type	Keyword	Modifier	Example
Mandatory	Speech	pov*	There was some *poverty* of *speech* and content of thought.
Optional	hallucinat*	Audit*	For past 1 week has been having *auditory* command *hallucinations* telling him to kill himself and also suicidal ideation*.*
Optional	hallucinat*		These *hallucinations* are sometimes in a kind of shadow form shaped like a man I call ‘David’ and ‘James’.
None	Rapport		When she was last seen at her CPA on XXXXX by Specialist Registrar Dr XXXXX, ZZZZZ presented as well kempt with good eye contact and *rapport*.

### Information extraction with TextHunter

TextHunter is an NLP information extraction suite developed jointly by SLaM and the Institute of Psychiatry, Psychology & Neuroscience at King's College London.^30^ Its principle purpose is to provide an interface to accomplish three tasks required to extract concepts from free text:

1. find instances of a concept in a database of documents using regular expression style matching of keywords;
2. provide an efficient interface to allow human annotators to label a portion of the sentences containing the concept instances in order to develop a gold standard and training corpora;
3. attempt to construct an appropriate support vector machine (SVM) language model of the concept, and validate it with the gold standard corpus.

Briefly, TextHunter is built around the ConText algorithm^31^ and the GATE framework Batch Learning plugin, a machine learning framework which in turn uses the LibSVM java library.^32^ A SVM is a machine learning methodology that maps the features of human labelled input training data instances into vector space. Within this space, a learning algorithm is applied to construct a hyperplane, which attempts to accurately differentiate the different training instances based on their labels. Once this hyperplane is ‘learnt’, the model can be applied to new, unseen instances to predict the label that should be assigned. TextHunter uses bag-of-words features such as keywords, surrounding word tokens and part-of-speech tags in conjunction with knowledge engineering features generated from ConText to build a sentence classifier. A full description of its workings is described in ref. 30. In this analysis, we used V.3.0.6 of TextHunter.

### Annotation of SMI symptom concepts

In order to produce annotation guidelines to ensure consistent, high-quality gold standard and training data, we developed annotation guidelines based around internal, iterative discussions. Generally, we defined a relevant instance as a mention of a symptom observed in a patient, without a grammatical negation. Owing to the large numbers of concepts addressed by this work, it was only feasible to double annotate 15 of the concepts to derive interannotator agreement (IAA) statistics. This was completed by either two psychiatrists or a psychiatrist and a trained research worker familiar with the construct.

To optimise the performance of the language models for the SMI cohort, we enriched our training corpus by selecting any text occurrence in CRIS (irrespective of the document type), relating to a patient who had received an SMI diagnosis, defined as schizophrenia (ICD-10 code F20x), schizoaffective disorder (F25x) or bipolar disorder (F31x). This diagnosis information came from structured fields in the source EHR, which are completed by clinicians during the normal course of care by means of selecting an appropriate ICD-10 code; these were supplemented by a separate NLP application^33^ ^34^ which returns searchable text strings associated with diagnostic statements in text fields. In UK NHS Mental Health Trusts, recording of diagnosis is effectively mandatory, but recorded diagnoses themselves have no financial implications for trusts (eg, are not used for any billing purposes).

An independent set of gold standard data were also created for each symptom to assess the performance of each model. This was derived in the same manner as the training data.

For training and gold standard data, a relevant instance of a symptom was labelled as ‘positive’, (such as ‘the patient had poverty of speech’) whereas irrelevant or negated instances (such as ‘today I examined the patient for poverty of speech…’ or ‘the patient did not have poverty of speech’) were labelled as ‘negative’ to create a binary classification problem (for the special case of the ‘negative symptoms’ construct, this was annotated as positive when described as present (eg, ‘he experiences severe negative symptoms’) and negative when absent (eg, ‘there was no evidence of negative symptoms’)). The training data were then used in 10-fold cross validation to estimate the optimal SVM model parameters using the features provided by TextHunter (see above). An instance was considered correctly classified if the sentence containing the human label of ‘positive’ or ‘negative’ and symptom type matched the model-generated label and symptom type. Subclassifications of classes based on any modifiers that were present were not evaluated in this work. Finally, we validated the optimised models against our gold standard data. We arbitrarily decided that the gold standard for each concept should contain a minimum of 100 ‘positive’ mentions, in order to derive precision, recall and F1 measures for the ‘positive’ class.

Owing to the tendency of a given set of clinical notes to repeat certain pieces of information over time, EHRs offer multiple opportunities to bolster recall (eg, via the reassessment of symptoms across multiple visits). For this reason, we favoured precision over recall as the more desirable performance metric. We applied SVM confidence margin filters to increase precision where acceptable losses to recall were possible. If performance was deemed to be poor, we attempted to improve the model by adding further training data, in some cases using TextHunter's active learning capability. In addition, we evaluated the accurate identification of the negation status of each symptom between TextHunter + ConText rules versus the ConText negation feature in isolation.

### Descriptive statistics of SMI distribution among SMI and non-SMI cohorts

A cohort of 18 761 patients was selected from CRIS, dating from the inception of electronic records in SLaM in 2006 to November 2014, all of whom had received an SMI diagnosis as defined above at any point during that period. For a negative control, we also selected a cohort of 57 999 patients that had received a non-SMI diagnosis, defined as the assignment of an ICD-10 code of F32 (depressive episode), F33 (recurrent depressive disorder, F40–F48 (neurotic, stress-related and somatoform disorders) or F60 (personality disorder) in the same period. F32.3 (severe depressive episode with psychotic symptom) and F33.3 (recurrent depressive disorder, current episode severe with psychotic symptoms) were excluded from the non-SMI cohort so as to not overlap with our SMI group. The NLP models were applied to a corpus of documents labelled as discharge summaries linked to these cohorts, and descriptive statistics were collected from the results.

## Results

### Interannotator agreement and model validation

In total, 50 different symptoms were chosen, and a total of 37 211 instances of symptoms were annotated from 32 767 documents to create gold standards and training data specific to each symptom. An additional 2950 instances across 15 symptoms were double annotated (table 3), yielding an average Cohen's κ of 0.83.

**Table 3:** Interannotator agreement scores

Project	Instances	Observed agreement	Cohen's κ
Catatonic syndrome	232	0.88	0.65
Diminished eye contact	362	1.00	1.00
Echolalia	98	0.96	0.89
Echopraxia	93	0.99	0.98
Elation	299	0.95	0.87
Euphoria	318	0.88	0.75
Grandiosity	293	0.94	0.84
Hallucinations	137	0.91	0.81
Immobility	98	0.90	0.79
Insomnia	291	0.93	0.69
Irritability	97	0.89	0.65
Mannerisms	89	0.89	0.73
Perseverance	99	0.97	0.93
Stupor	98	0.92	0.82
Waxy flexibility	135	0.95	0.89

Across all 50 symptoms, the average count of instances per gold standard was 202. Of the 50 symptoms for which we attempted to build models, four performed poorly (loosening of associations, stereotypy, low mood and poor motivation). Two symptoms were so rare (catalepsy, echopraxia) that it was practical to annotate all detected mentions of the keywords by hand. One symptom (mutism) achieved an acceptable performance based on the mention of the symptom keyword alone. Of the remaining 43 symptoms, the hybrid model produced a precision of at least 85% in 38 symptoms, compared with 23 symptoms using the ConText negation model alone. The precision, recall and F1 metrics of each modelled symptom for individuals with an SMI diagnosis are listed in online supplementary table 1. Summary statistics aggregated across all symptoms for each approach are presented in table 4.

**Table 4:** Comparison of the hybrid approach and context alone across all symptoms (excluding catalepsy, echopraxia and mutism in SMI cohort)

Statistic	Model	P%	R%	F1
Mean	ConText + ML	83	78	0.80
	ConText	71	97	0.79
Median	ConText + ML	90	85	0.88
	ConText	84	98	0.91

SMI, severe mental illness.

10.1136/bmjopen-2016-012012.supp1 supplementary table

### Analysis of discharge summaries

**Figure 1:**
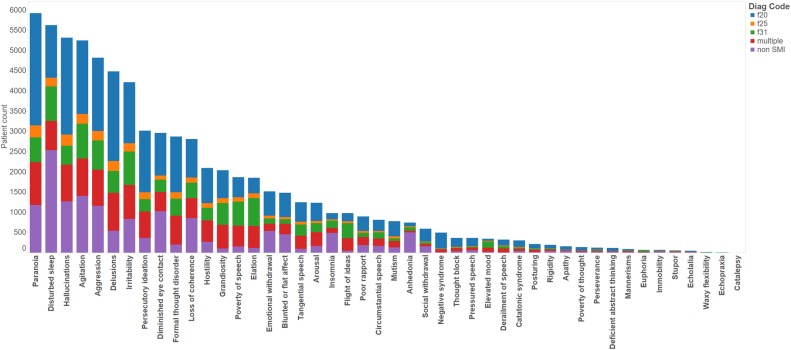
Distribution of symptoms by SMI ICD diagnosis. ICD, International Classification of Diseases; SMI, severe mental illness.

**Figure 2:**
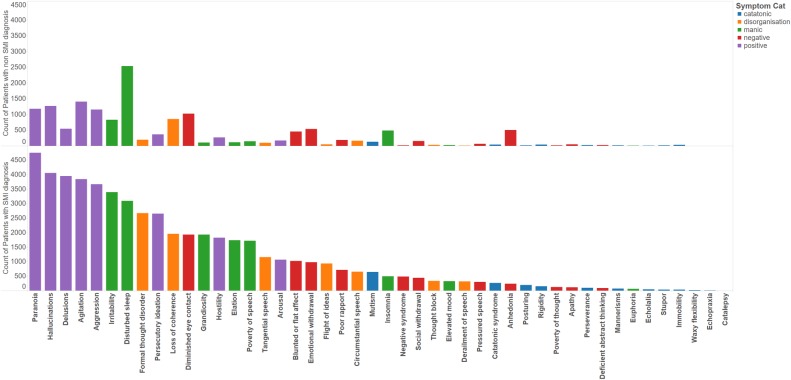
Distribution of symptoms by symptom classes.

Of the 18 761 patients in our SMI cohort, we were able to identify at least one labelled discharge summary for a subset of 7962 patients, to generate a corpus of 23 128 discharge summaries. For the 57 999 patients in our non-SMI cohort, we identified 13 496 discharge summaries for a subset of 7575 patients. The 43 NLP models were applied to the SMI and non-SMI corpora, which returned a total of 171 523 symptoms in 17 902 (77%) summaries across 6 920 (87%) patients in the SMI cohort and 31 769 symptoms in 7 259 (54%) summaries across 4540 (60%) patients in the non-SMI cohort (when combined with additional data from the three symptoms where NLP was not necessary). For succinctness, we grouped the symptoms into five semantic types, as described in table 5. The most common types were the positive symptoms (9662 patients) and the least common were the catatonic symptoms (1363 patients) (table 6). In figures 1 and 2, we plot bar charts of the counts of unique patients exhibiting each symptom, coloured by the original ICD-10 diagnosis and symptom domains respectively. In the SMI cohort, the counts of patients exhibiting the various symptoms follow an approximately Poisson distribution, with the prevalence of each symptom ranging from very common (paranoia, 59%) to very rare (catalepsy, >1%) (figure 2). In the negative control group, appreciable counts were also observed for many of the symptoms, with disturbed sleep the most common, followed by paranoia, hallucinations, agitation, aggression, diminished eye contact and loss of coherence.

**Table 5:** Symptom groupings

Domain	Symptoms
Positive	Agitation, aggression, arousal, hostility, delusions, hallucinations, paranoia, persecution
Negative	Diminished eye contact, blunted or flat affect, emotional withdrawal, social withdrawal, abstract thinking, poor rapport, apathy, anhedonia, poverty of speech, poverty of thought, negative syndrome
Disorganisation	Circumstantial speech, reduced coherence, formal thought disorder, thought block, tangential speech, derailment, flight of ideas
Manic	Elevated mood, disturbed sleep, insomnia, euphoria, pressured speech, irritability, elation, grandiosity
Catatonic	Mannerism, rigidity, posturing, perseverance, stupor, waxy flexibility, immobility, echolalia, mutism, catalepsy, echopraxia

**Table 6:** Counts of patients by symptom groups and ICD-10 diagnosis

Diagnosis	Catatonic	Disorganisation	Manic	Negative	Positive
F20—Schizophrenia	630	2076	2490	1903	3518
F25—Schizoaffective	71	252	370	206	432
F31—Bipolar	139	878	1316	529	1264
Multiple	268	987	1193	724	1331
Non-SMI	255	1182	3097	1984	3117
Total	1363	5375	8466	5346	9662

ICD-10, International Classification of Diseases, Tenth Revision; SMI, severe mental illness.

## Discussion

Using a large mental health EHR data resource, we were able to generate an extensive NLP-derived profiling of symptomatology in SMI, albeit limited to English language discharge summaries from patients who had received an ICD-10 SMI diagnosis. This yielded high volumes of novel information on 46 symptoms across five key domains. Comparable projects that we are aware of in mental healthcare have been the characterisation of diagnostic profiles in a Danish Psychiatric Case Register,^35^ and the use of NLP-derived symptoms to assist in the diagnosis of bipolar disorder in the US EHRs.^36^

The aspiration of the ‘CRIS-CODE’ project is to use NLP to offer comprehensive profiling from the mental health electronic record of symptoms and of interventions, outcomes and other relevant contextual factors currently only available from text fields. Our choice of symptoms for this initial phase of information extraction was arbitrary, based on the pragmatic criteria previously stated, and not intended to be comprehensive. In addition, the categories applied to group symptoms also have to be considered as arbitrary, albeit consistent with dimensions proposed by other authors, and need further empirical evaluation. The results of the IAA exercise across 15 symptoms suggest general good agreement in our definition of symptom instances, meaning that these concepts were generally well defined and understood among clinicians. A limitation of our IAA validation approach was that we did not sample across all symptoms, as the resource overhead to train all annotators in all concepts was prohibitive. Regarding TextHunter model effectiveness, our results indicate that good information extraction performance could be achieved in the majority of SMI symptom concepts that we attempted, using the standard hybrid approach of ConText rules and machine learning offered by TextHunter. This suggests that future work to expand on this list should also be a tractable problem with this methodology. We were also able to demonstrate that the hybrid approach of combining ConText and ML generally performs favourably compared with ConText in isolation, when precision is favoured over recall, although ConText in isolation outperforms the hybrid approach if recall is favoured. As ConText was designed with medical records from the USA in mind, it is possible that the differences in medical language between British English and American English may account for the relatively low precision of ConText alone on UK medical records. However, it also conforms to the expectation that generic NLP systems for IE have limitations when applied to specific phenomena in mental health symptomatology compared with ML models trained for a specific purpose using expert clinical annotations.

In the case of some symptoms, neither the hybrid method nor ConText alone was able to deliver adequate performance. This is most likely due to the common occurrence in other contexts of the keywords used to describe instances of these symptoms, and the difficulty in disambiguating between their general use and their clinical use. For example, it is very common for a caregiver to describe a patient's ‘motivation’ in a variety of contexts, and differentiating a specific clinical symptom of ‘poor motivation’ will likely require alternative approaches. A related example might also be the variety of terms used to describe low mood, and its proximity in standard mental state examination text to statements concerning lowered or depressed *affect*—a similar but different entity (‘mood’ conventionally referring to a patient's reported experience of their emotional status; ‘affect’ to the clinician's observation of the same). It is likely that our approach of enriching the training data via selecting text from individuals with an SMI diagnosis failed to provide sufficient feature diversity for the SVMs to differentiate between relevant and irrelevant instances. Future work might address this by more detailed exploration of the common clinical language used to describe the failed concepts, in order to use knowledge engineering to derive more valuable features than a simple bag-of-words approach can yield.

An important consideration is that we were only able to identify a minimum of one symptom in 87% of patients with SMI from the corpus of documents sampled, suggesting additional recall improvements should be possible. Underestimation of symptoms may have occurred for several reasons. First, we did not specify a minimum length of treatment in our inclusion criteria, so relatively new patients with sparse documentation may not yet have any symptoms registered in their record. Second, our predilection for precision over recall in tuning our models may have reduced the probability of detection. Third, our list of symptoms was not comprehensive and may have missed some aspects of psychosis presentation—either because of different symptoms which were missed, or because of target symptoms which were described in non-standard language (eg, ‘hearing voices’ rather than ‘auditory hallucination’)—although as per our methodological reasons regarding the use of synonyms, including non-standard terms may introduce additional uncertainty as to the author’s intended meaning. It is also possible that the SMI diagnosis had been first recorded at an earlier presentation and that some patients were now presenting with different sets of symptoms not currently captured (eg, people with bipolar disorder who were currently depressed, or people with previous schizophrenia currently receiving care for alcohol or drug dependence). Further in-depth exploration of text fields is warranted in the sample with no symptoms identified from the current list, to clarify the nature of symptoms and presentations reported; such an exercise would be feasible in CRIS, but was felt to be beyond the scope of this paper. Fourth, the descriptive data were restricted to a specific corpus of documents described as discharge summaries. Discharge summaries might be considered the most ‘valuable’ clinical documents in NLP tasks because of their emphasis on detail and accuracy, and the tendency for institutions to encourage clinicians to use standard language in their authorship. However, it is possible that symptoms may be recorded in other areas of the record that would not have been captured by our approach. To maximise recall by including additional document types raises new questions for NLP tasks such as the importance of an author's profession and temporal aspects relating to the amount of patient/clinician contact. Finally, sufficiency of the source may be in question—for example, the CRIS database does not currently have the capacity to process scanned images of text documents (as opposed to formats such as Microsoft Word) and these images of text documents are known to make up approximately a third of all uploaded files to the clinical database. Alternatively, discharge summaries that were mislabelled as another document class at the point of upload also would not have been included in our analysis. A document classification approach may assist here.

Appreciable prevalences of many of the symptoms in the group with a non-SMI diagnosis are not unexpected, given the extent to which mental health symptoms are recognised to cross diagnostic categories—one of the factors behind CRIS-CODE's objectives. For example, sleep disturbance and diminished eye contact are common features of depressive disorder, and agitation and aggression are similarly non-specific. The common occurrence of paranoia and hallucinations would benefit from more detailed future evaluation, although might reflect early psychotic syndromes which had not yet attracted an SMI (or depressive psychosis) diagnosis, or else unrelated phenomena (eg, non-specific hallucinatory experiences accompanying sleep disturbance) or inappropriately applied terminology (eg, paranoia used to describe non-delusional hostility or suspiciousness).

## Conclusion

The primary purpose of the developments described was to improve the depth of information available on patients with these disorders represented on healthcare datasets, as these information resources frequently contain little information beyond a diagnosis. The case for identifying symptoms of SMI as a source of data for mental health research is driven by widely recognised deficiencies of diagnostic categories alone for capturing mental disorders or providing adequate classes with which to cluster groups of patients for research or intervention. This is compounded by the lack of an instrument to capture symptomatology, as most research instruments would be considered overly cumbersome for routine clinical application outside specialist services. Furthermore, even if a fully structured instrument was identified as acceptable for use in initial assessment, obtaining real-time repeated measurements would present even more substantial challenges. The situation currently in mental health EHRs is that symptom profiles have been ‘invisible’ when it comes to deriving data for research, service development or clinical audit. Given that they are key determinants of interventions received and outcomes experienced, this has been a major deficiency. We therefore hope that the outputs of this project will offer the tools/techniques to use the large amounts of SMI symptomatology data contained within EHR systems, and provide new insight into the value of using SMI symptoms as predictors of a range of outcome measures. Although we did not seek to extend our analyses beyond simple descriptions of distributions, these strongly indicate that symptoms cross diagnostic groupings—for example, indicating that affective symptoms were not restricted to bipolar disorder. This is consistent with other reported findings from CRIS on mood instability which also cut across ‘affective’ and ‘non-affective’ psychosis^37^ and which suggests that symptom dimensions rather than traditional diagnostic groupings may be a more valid approach to investigating aetiology and outcome in psychosis.
