# Supplementary material for: Natural language processing to extract symptoms of severe mental illness from clinical text: the Clinical Record Interactive Search Comprehensive Data Extraction (CRIS-CODE) project
Source: BMJ Open. 2017 Jan 17;7(1):e012012. doi: 10.1136/bmjopen-2016-012012 (PMC5253558; doi:10.1136/bmjopen-2016-012012)
Supplement: supplementary table [file bmjopen-2016-012012supp_table.pdf]

NLP symptom model performance against gold standards (P=precision, R=recall, 95% confidence intervals in parenthesis)

Symptom	Model	Training Instances	TP	TN	FP	FN	P %	R %	F1
Aggression	ConText + ML	318	130	41	14	9	90 (+/- 5)	94 (+/- 4)	0.92
	ConText		135	22	33	4	80 (+/- 7)	97 (+/- 3)	0.88
Agitation	ConText + ML	296	170	11	10	1	94 (+/- 3)	99 (+/- 1)	0.97
	ConText		168	14	7	3	96 (+/- 3)	98 (+/- 2)	0.97
Anhedonia	ConText + ML	369	71	55	3	13	96 (+/- 4)	85 (+/- 8)	0.90
	ConText		83	52	6	1	93 (+/- 5)	99 (+/- 2)	0.96
Apathy	ConText + ML	287	90	20	4	23	96 (+/- 4)	80 (+/- 7)	0.87
	ConText		109	3	21	4	84 (+/- 7)	96 (+/- 3)	0.90
Arousal	ConText + ML	298	139	20	7	31	95 (+/- 3)	82 (+/- 6)	0.88
	ConText		169	6	21	1	89 (+/- 5)	99 (+/- 1)	0.94
Blunted or flat affect	ConText + ML	503	37	164	2	29	95 (+/- 5)	56 (+/- 12)	0.70
	ConText		66	18	148	0	31 (+/- 11)	100 (+/-0)	0.47
Catalepsy[1]	~								
Catatonic syndrome	ConText + ML	683	118	10	9	9	93 (+/- 4)	93 (+/- 4)	0.93
	ConText		122	6	13	5	90 (+/- 5)	96 (+/- 3)	0.93
Circumstantial speech	ConText + ML	271	79	323	12	17	87 (+/- 7)	82 (+/- 8)	0.84
	ConText		96	23	312	0	24 (+/- 8)	100 (+/-)	0.38
Deficient abstract thinking	ConText + ML	597	50	101	3	53	94 (+/- 4)	49 (+/- 1)	0.64
	ConText		100	26	78	3	56 (+/- 1)	97 (+/- 3)	0.71
Delusions	ConText + ML	677	99	28	3	8	97 (+/- 3)	93 (+/- 5)	0.95
	ConText		105	24	7	2	94 (+/- 5)	98 (+/- 3)	0.96
Derailment of speech	ConText + ML	280	104	15	5	4	95 (+/- 4)	96 (+/- 4)	0.96
	ConText		108	9	11	0	91 (+/- 5)	100 (+/-)	0.95
Diminished eye contact	ConText + ML	661	84	296	18	23	82 (+/- 7)	79 (+/- 8)	0.80
	ConText		86	4	310	21	22 (+/- 8)	80 (+/- 8)	0.34
Disturbed sleep	ConText + ML	750	58	27	10	11	85 (+/- 8)	84 (+/- 9)	0.85
	ConText		64	10	27	5	70 (+/- 11)	93 (+/- 6)	0.80

Symptom	Model	Training Instances	TP	TN	FP	FN	P %	R %	F1
Echolalia	ConText + ML	475	74	10	6	0	93 (+/- 6)	100 (+/- 0)	0.96
	ConText		73	7	9	1	89 (+/- 7)	99 (+/- 3)	0.94
Echopraxia[1]	~								
Elation	ConText + ML	335	177	39	20	11	90 (+/- 4)	94 (+/- 3)	0.92
	ConText		188	33	26	0	88 (+/- 5)	100 (+/- 0)	0.94
Elevated mood	ConText + ML	947	97	59	16	31	86 (+/- 6)	76 (+/- 7)	0.80
	ConText		125	37	38	3	77 (+/- 7)	98 (+/- 3)	0.86
Emotional withdrawal	ConText + ML	574	74	197	11	37	87 (+/- 6)	67 (+/- 9)	0.76
	ConText		110	47	161	1	41 (+/- 9)	99 (+/- 2)	0.58
Euphoria	ConText + ML	288	102	26	11	20	90 (+/- 5)	84 (+/- 7)	0.87
	ConText		120	19	18	2	87 (+/- 6)	98 (+/- 2)	0.92
Flight of ideas	ConText + ML	273	104	26	6	1	95 (+/- 4)	99 (+/- 2)	0.97
	ConText		103	29	3	2	97 (+/- 3)	98 (+/- 3)	0.98
Formal thought disorder	ConText + ML	605	97	139	15	6	87 (+/- 7)	94 (+/- 5)	0.90
	ConText		101	141	13	2	89 (+/- 6)	98 (+/- 3)	0.93
Grandiosity	ConText + ML	381	179	34	21	2	90 (+/- 4)	99 (+/- 2)	0.94
	ConText		176	34	21	5	89 (+/- 4)	97 (+/- 2)	0.93
Hallucinations	ConText + ML	1013	108	42	6	3	95 (+/- 4)	97 (+/- 3)	0.96
	ConText		110	44	4	1	96 (+/- 3)	99 (+/- 2)	0.98
Hostility	ConText + ML	581	163	13	18	2	90 (+/- 5)	99 (+/- 2)	0.94
	ConText		162	13	18	3	90 (+/- 5)	98 (+/- 2)	0.94
Immobility	ConText + ML	718	30	19	7	6	81 (+/- 13)	83 (+/- 12)	0.82
	ConText		36	4	22	0	62 (+/- 16)	100 (+/- 0)	0.77
Insomnia	ConText + ML	234	68	2	9	6	88 (+/- 7)	92 (+/- 6)	0.90
	ConText		73	3	8	1	90 (+/- 7)	99 (+/- 3)	0.94
Irritability	ConText + ML	632	146	16	23	12	86 (+/- 5)	92 (+/- 4)	0.89
	ConText		158	15	24	0	87 (+/- 5)	100 (+/- 0)	0.93
Loosening of associations	ConText + ML	353	0	1145	0	141	0 (+/- 0)	0 (+/- 0)	0.00
	ConText		141	110	1035	0	12 (+/- 5)	100 (+/- 0)	0.21

Symptom	Model	Training Instances	TP	TN	FP	FN	P %	R %	F1
Loss of coherence	ConText + ML	601	171	25	31	35	85 (+/- 5)	83 (+/- 5)	0.84
	ConText		203	4	52	3	80 (+/- 6)	99 (+/- 2)	0.88
Low mood	ConText + ML	879	0	35	0	21	0 (+/- 0)	0 (+/- 0)	0.00
	ConText		21	2	33	0	39 (+/- 21)	100 (+/- 0)	0.56
Mannerisms	ConText + ML	689	52	73	17	13	75 (+/- 1)	80 (+/- 1)	0.78
	ConText		62	36	54	3	53 (+/- 12)	95 (+/- 5)	0.69
Mutism[2]	Keyword		93	1	9	4	91 (+/- 6)	96 (+/- 4)	0.93
Negative syndrome	ConText + ML	150	114	12	11	12	91 (+/- 5)	90 (+/- 5)	0.91
	ConText		125	7	16	1	89 (+/- 6)	99 (+/- 2)	0.94
Paranoia	ConText + ML	263	144	10	8	3	95 (+/- 4)	98 (+/- 2)	0.96
	ConText		144	13	5	3	97 (+/- 3)	98 (+/- 2)	0.97
Persecutory ideation	ConText + ML	297	153	14	7	2	96 (+/- 3)	99 (+/- 2)	0.97
	ConText		152	15	6	3	96 (+/- 3)	98 (+/- 2)	0.97
Perseverance	ConText + ML	728	45	111	3	14	94 (+/- 6)	76 (+/- 11)	0.84
	ConText		59	11	103	0	36 (+/- 12)	100 (+/-)	0.53
Poor motivation	ConText + ML	753	0	249	0	116	0 (+/- 0)	0 (+/- 0)	0.00
	ConText		105	12	237	11	31 (+/- 8)	91 (+/- 5)	0.46
Poor rapport	ConText + ML	803	50	493	2	56	96 (+/- 4)	47 (+/- 1)	0.63
	ConText		90	6	489	16	16 (+/- 7)	85 (+/- 7)	0.26
Posturing	ConText + ML	1709	94	312	9	39	91 (+/- 5)	71 (+/- 8)	0.80
	ConText		131	46	275	2	32 (+/- 8)	98 (+/- 2)	0.49
Poverty of speech	ConText + ML	373	172	54	17	18	91 (+/- 4)	91 (+/- 4)	0.91
	ConText		189	33	38	1	83 (+/- 5)	99 (+/- 1)	0.91
Poverty of thought	ConText + ML	368	97	7	2	8	98 (+/- 3)	92 (+/- 5)	0.95
	ConText		98	6	3	7	97 (+/- 3)	93 (+/- 5)	0.95
Pressured speech	ConText + ML	377	268	11	14	23	95 (+/- 2)	92 (+/- 3)	0.94
	ConText		285	8	17	6	94 (+/- 3)	98 (+/- 2)	0.96
Rigidity	ConText + ML	1331	43	90	11	14	80 (+/- 1)	75 (+/- 11)	0.77
	ConText		56	41	60	1	48 (+/- 13)	98 (+/- 3)	0.65
Social withdrawal	ConText + ML	307	137	33	24	18	85 (+/- 6)	88 (+/- 5)	0.87
	ConText		153	4	53	2	74 (+/- 7)	99 (+/- 2)	0.85

Symptom	Model	Training Instances	TP	TN	FP	FN	P %	R %	F1
Stereotypy	ConText + ML	1397	0	72	0	64	0 (+/- 0)	0 (+/- 0)	0.00
	ConText		63	23	49	1	56 (+/- 12)	98 (+/- 3)	0.72
Stupor	ConText + ML	567	84	21	12	11	88 (+/- 7)	88 (+/- 6)	0.88
	ConText		93	8	25	2	79 (+/- 8)	98 (+/- 3)	0.87
Tangential speech	ConText + ML	250	101	0	7	0	94 (+/- 5)	100 (+/- 0)	0.97
	ConText		99	2	5	2	95 (+/- 4)	98 (+/- 3)	0.97
Thought block	ConText + ML	377	85	32	9	41	90 (+/- 5)	67 (+/- 8)	0.77
	ConText		124	19	22	2	85 (+/- 6)	98 (+/- 2)	0.91
Waxy flexibility	ConText + ML	453	66	91	6	13	92 (+/- 6)	84 (+/- 8)	0.87
	ConText		76	51	46	3	62 (+/- 11)	96 (+/- 4)	0.76

1. All instances annotated. 2. Keyword only – no NLP applied
